# PAM-1: an antimicrobial peptide with promise against ceftazidime-avibactam resistant *Escherichia coli* infection

**DOI:** 10.3389/fmicb.2024.1291876

**Published:** 2024-04-30

**Authors:** Yijia Han, Yi Zhang, Xiaodong Zhang, Zeyu Huang, Jingchun Kong, Xiuxiu Wang, Lijiang Chen, Yue Wang, Jianming Cao, Tieli Zhou, Mo Shen

**Affiliations:** ^1^Department of Clinical Laboratory, Key Laboratory of Clinical Laboratory Diagnosis and Translational Research of Zhejiang Province, The First Affiliated Hospital of Wenzhou Medical University, Wenzhou, China; ^2^School of Laboratory Medicine and Life Science, Wenzhou Medical University, Wenzhou, China

**Keywords:** *E* *scherichia coli*, biofilm, CZA -resistant, PAM-1, antimicrobial peptide

## Abstract

**Introduction:**

Antibiotic misuse and overuse have led to the emergence of carbapenem-resistant bacteria. The global spread of resistance to the novel antibiotic combination ceftazidime-avibactam (CZA) is becoming a severe problem. Antimicrobial peptide PAM-1 offers a novel approach for treating infections caused by antibiotic-resistant bacteria. This study explores its antibacterial and anti-biofilm activities and mechanisms against CZA-resistant *Escherichia. Coli (E. coli)*, evaluating its stability and biosafety as well.

**Methods:**

The broth microdilution method, growth curve analysis, crystal violet staining, scanning electron microscopy, and propidium iodide staining/N-phenyl-1-naphthylamine uptake experiments were performed to explore the antibacterial action and potential mechanism of PAM-1 against CZA-resistant E. coli. The biosafety in diverse environments of PAM-1 was evaluated by red blood cell hemolysis, and cytotoxicity tests. Its stability was further assessed under different temperatures, serum concentrations, and ionic conditions using the broth microdilution method to determine its minimum inhibitory concentration (MIC). Galleria mellonella infection model and RT-qPCR were used to investigate the in vivo antibacterial and anti-inflammatory effects.

**Results and discussion:**

*In vitro* antibacterial experiments demonstrated that the MICs of PAM-1 ranged from 2 to 8 μg/mL, with its effectiveness sustained for a duration of 24 h. PAM-1 exhibited significant antibiofilm activities against CZA-resistant *E. coli* (*p* < 0.05). Furthermore, Membrane permeability test revealed that PAM-1 may exert its antibacterial effect by disrupting membrane integrity by forming transmembrane pores (*p* < 0.05). Red blood cell hemolysis and cytotoxicity tests revealed that PAM-1 exerts no adverse effects at experimental concentrations (*p* < 0.05). Moreover, stability tests revealed its effectiveness in serum and at room temperature. The Galleria mellonella infection model revealed that PAM-1 can significantly improve the survival rate of *Galleria mellonella* (>50%)for *in vivo* treatment. Lastly, RT-qPCR revealed that PAM-1 downregulates the expression of inflammatory cytokines (*p* < 0.05). Overall, our study findings highlight the potential of PAM-1 as a therapeutic agent for CZA-resistant *E. coli* infections, offering new avenues for research and alternative antimicrobial therapy strategies.

## 1 Introduction

Global public health is increasingly threatened by antibiotic resistance, with carbapenem-resistant *Enterobacteriaceae* (CRE) posing a significant challenge by elevating infection rates and mortality (Satlin et al., 2017; van Duin et al., 2020). The limited effectiveness of traditional antibiotics led to the 2015 FDA (Food and Drug Administration) approval of ceftazidime-avibactam (CZA), a novel combination of a cephalosporin and a β-lactamase inhibitor. This innovative combination exhibits *in vitro* activity against *Enterobacterales* harboring β-lactamases of Ambler class A [including extended-spectrum β-lactamases (ESBLs) and *Klebsiella pneumoniae* carbapenemases (KPC)], class C (AmpC cephalosporinases), and some class D enzymes (e.g., OXA-48-type, many of which also possess ESBLs) (Morrill et al., 2015; Falcone et al., 2016; Livermore et al., 2018; Zhang et al., 2018). However, the subsequent global increase in CZA usage has gradually increased CZA resistance, with new cases of CZA-resistant *E. coli* strains being frequently reported (Shields et al., 2016, 2017; Hernández-García et al., 2021). Therefore, developing novel antimicrobial drugs to combat CZA-resistant *E. coli* infections is urgently warranted.

Antimicrobial peptides (AMPs), an intrinsic component of the innate immune system in several animals, confer protection against foreign microorganisms and infectious threats (Pasupuleti et al., 2012). In general, these peptides are short and positively charged and exhibit broad-spectrum antibacterial activity as well as effects on biofilms (Spencer et al., 2018; Luo and Song, 2021). Furthermore, AMPs can regulate inflammatory responses and promote wound healing (Nguyen et al., 2020; Thapa et al., 2020). To enhance the application of antimicrobial peptides (AMPs) in clinical antimicrobial treatments, researchers are actively engaged in identifying and screening AMPs that not only demonstrate potent antimicrobial effects but also exhibit a high level of safety.

PAM-1, an antimicrobial peptide sourced from the cathelicidin gene family in the platypus genome, exhibits significant antibacterial effects against standard strains of *Bacillus subtilis*, *Staphylococcus aureus*, *Streptococcus uberis*, *Streptococcus pyogenes*, *E. coli*, *Salmonella cholerae*, and *Pseudomonas aeruginosa* (Wang et al., 2011). Through bioinformatics analysis, it is predicted that PAM-1 possesses a wide-ranging antibacterial activity against various bacteria. However, regrettably, the antibacterial and antibiofilm activities and the mechanisms of action of PAM-1 against clinical strains remain unelucidated in previous studies.

In the present study, a series of experiments were conducted to investigate the antibacterial, antibiofilm, and anti-inflammatory activities of PAM-1 against CZA-resistant *E. coli* strains. The potential mechanisms by which PAM-1 exerts its antibacterial effects were elucidated, and its stability and safety were thoroughly assessed. Additionally, the *in vivo* efficacy of PAM-1 was evaluated using the *Galleria mellonella* (*G. mellonella*) infection model. The findings of this study suggest that PAM-1 holds promise as a novel antibacterial agent for combating CZA-resistant *E. coli* infections.

## 2 Methods

### 2.1 AMP and antibiotics

PAM-1 (95.66%, 100 mg) was synthesized by Nanjing Yuanpeptide Biotechnology Co., Ltd. (Nanjing, China). The amino acid sequence of PAM-1 is RTKRRIKLIKNGVKKVKDILKNNNIIILPGSNEK (Wang et al., 2011). To prepare PAM-1 for the experiments, it was dissolved in double distilled water and stored at −20°C. Ceftazidime (≥ 99%), avibactam (≥ 99%), meropenem (MEM, ≥ 98.0%), and imipenem (IPM, ≥ 98.0%) used in this study were purchased from Wenzhou Kangtai Biotechnology Co., Ltd. (Zhejiang, China).

### 2.2 Characterization of PAM-1

PAM-1 was analyzed using a predictive analysis tool in ProtParam of Expasy (https://web.expasy.org/protparam/). The physical and chemical properties of PAM-1, including net charge and relative molecular mass, were determined. The antibacterial activity of PAM-1 was predict using the online software CAMPR3 (http://www.camp3.bicnirrh.res.in/).

### 2.3 Bacteria isolates and growth conditions

Six CZA-susceptible *E. coli* and six CZA-resistant *E. coli* isolates were selected from the First Affiliated Hospital of Wenzhou Medical University. All isolates were identified using MALDI-TOF MS (Quintilla et al., 2018; Ashfaq et al., 2022) (BioMerieux, France). These clinically relevant *E. coli* strains were preserved by freezing them at −80°C in Luria–Bertani (LBT, hermo Fisher Scientific, America) broth containing 30% glycerol. *E. coli* ATCC 25922 was used as the quality control strain.

### 2.4 Enzyme types of the 12 *Escherichia coli* strains

DNA was extracted from the 12 *E. coli* strains using a bacterial genome extraction kit (Solarbio, Beijing, China) according to the manufacturer’s instructions (Zeng et al., 2022). The resistant determinants, including carbapenem genes (*bla*_KPC_, *bla*_NDM_, *bla*_IMP_, *bla*_VIM_, *bla*_OXA − 23_, and *bla*_OXA − 48_) and ESBL genes (*bla*_SHV_, *bla*_TEM_, *bla*_CTX − M − 1_, *bla*_CTX − M − 9_, and *bla*_CTX − M − 14_), were examined via PCR using specific primers (Supplementary Table S1). Subsequently, the positive PCR products were sequenced and aligned using the BLAST tool in NCBI (https://blast.ncbi.nlm.nih.gov/Blast.cgi).

### 2.5 Antimicrobial activity of PAM-1, CZA, and carbapenems against *Escherichia coli*

The broth microdilution method was used to determine the minimum inhibitory concentrations (MICs) of CZA, MEM, IPM, and PAM-1 against the 12 *E. coli* strains (García-Cobos et al., 2008). The 0.5 McFarland bacterial suspension was 1:100 diluted by Mueller Hinton broth (MHB, Thermo Fisher Scientific, America). Then, 100 μL of the bacterial solution was added to a 96-well microplate containing various concentrations of the antibiotics: CZA, MEM, IPM, and PAM-1. The microplates were incubated at 37°C for 16–18 h. The results were interpreted based on the 2020 Clinical and Laboratory Standards Institute guidelines (CLSI). MIC was defined as the lowest concentration of an antibiotic that completely inhibited bacterial growth.

To determine the CRE (MBC), 100 μL of the bacterial suspension from the well corresponding to the MIC and three subsequent wells was collected and spread onto LB agar plates. Then, the plates were incubated at 37°C for 16–20 h. MBC was determined as the drug concentration that resulted in no bacterial colony formation on the plate.

Each experiment was independently repeated three times to ensure the accuracy and reproducibility of the results.

### 2.6 Growth curve analysis

The growth curve was determined according to a previously described method, with some modifications (Jayathilaka et al., 2021). The diluted *E. coli* culture was added to a 96-well plate along with PAM-1 at concentrations corresponding to its 1/2 MIC, 1 MIC, 2 MIC, and 4 MIC (ranging from 1–32 μg/mL). The plate was incubated at 37°C for 24 h. Bacterial growth was monitored by measuring the absorbance at 600 nm at different time intervals (0, 2, 4, 6, 8, 10, 12, and 24 h). Each experiment was repeated three times to ensure accuracy and reproducibility.

### 2.7 Biofilm formation and eradication tests

The effect of PAM-1 on biofilm formation and eradication was determined by performing crystal violet staining (O’Loughlin et al., 2013). The bacterial culture was added to a 96-well plate with the corresponding concentration of PAM-1. After incubation for 24 h at 37°C, the 96-well plate was gently washed with phosphate-buffered saline (PBS, Beijing Solarbio Science & Technology Co., Ltd., Beijing, China) to remove any planktonic bacteria. Then, the plate was allowed to naturally dry at room temperature. Subsequently, a 0.1% crystal violet solution was added to each well, followed by incubation at 37°C for 15 min. After washing the plate with PBS and allowing it to naturally dry, 200 μL of a solution containing 95% ethanol and 5% acetic acid was added to dissolve the crystal violet. Biofilm biomass was calculated by measuring the absorbance at 595 nm. Two separate tests were performed: one to evaluate the effect of PAM-1 on biofilm formation (where the drug was added before biofilm formation) and another to evaluate the eradication of mature biofilms (where the drug was added after biofilm formation). Each test was conducted three times, and each experiment within a test was repeated three times for reliability and consistency.

### 2.8 Scanning electron microscopy

The SEM was determined according to a previously described method, with some modifications (Zhang et al., 2019). Silicon wafers were placed in the wells and 10 μL of the bacterial suspension was added to 990 μL of MHB containing PAM-1. Then, the plates were incubated for 24 h. Thereafter, the wafers were cleaned three times with PBS, fixed with 2.5% glutaraldehyde, and dehydrated for 5 min using an ethanol concentration gradient (30, 50, 70, 80, 90, 95, and 100%). The final samples were air-dried, gold-sprayed, and observed under a scanning electron microscope (SU8010, Hitachi, Japan).

### 2.9 Membrane permeability test and ROS detection

To investigate the mechanism of antimicrobial activity of PAM-1, membrane permeability tests were conducted as described previously, with some modifications (Liu et al., 2017). The bacteria suspension was shaken in the medium to the logarithmic stage and divided into equal parts. Then, different concentrations of PAM-1 were added to the bacterial suspensionand was continued to be shaken for 2 h. The sample after centrifugation were resuspended with propidium iodide (PI, Beijing Solarbio Science & Technology Co., Ltd., Beijing, China) and N-phenyl-1-naphthylamine (NPN, Beijing Solarbio Science & Technology Co., Ltd., Beijing, China) solution and incubated in a water bath at 37°C for 30 min. Then, the samples were scanned at excitation wavelengths of 535 nm and 350 nm and emission wavelengths of 615 nm (PI) and 420 nm (NPN), respectively, using a microplate reader (BioTek Synergy NEO2, United States).

To detect reactive oxygen species (ROS), 500 μL of the bacterial suspension containing dichlorodihydrofluorescein diacetate (DCFH-DA, Shanghai Generay Biotech Co., Ltd) was incubated at 37°C for 60 min. After removing excess DCFH-DA probes, the cells were then treated with PAM-1 at 30°C for 2 h. Sterile distilled water was used as the blank control. All samples were scanned at an excitation wavelength of 488 nm and an emission wavelength of 535 nm using a microplate reader (BioTek Synergy NEO2). This assay aimed to investigate the mechanism of action of PAM-1. Each experiment was independently repeated three times.

### 2.10 Expression of proinflammatory cytokines

To further evaluate the effect of PAM-1 on the expression of proinflammatory factors, reverse transcription quantitative PCR (RT-qPCR) was performed using a previously described method, with slight modifications (de Campos et al., 2020). RAW 264.7(Procell Life Science and Technology Co., Ltd., China) cells were cultured overnight and Dulbecco’s modified Eagle medium (DMEM, Thermo Fisher Scientific, USA) supplemented with 10% fetal bovine serum (FBS, Gibco, USA) was used to culture the cells. The cells were washed three times with PBS, and the culture medium was discarded. Bacteria at a multiplicity of infection (MOI) of 10 were added to infect the cells, followed by treatment with different concentrations of PAM-1for 2 h. After removing the culture medium and washing with PBS, total RNA was extracted from RAW 264.7 cells using the Trizol total RNA extraction method.

Total RNA was extracted from RAW 264.7 cells using the Trizol total RNA extraction method (Tang et al., 2017; Trendel et al., 2019). After measuring the RNA concentration (DeNovix ultramicro-UV–visible spectrophotometer DS-11, USA), the extracted mRNA was converted into cDNA using the PrimeScript™ RT Reagent Kit according to the manufacturer’s instructions. The expression of the inflammatory factors interleukin (IL)-1β and tumor necrosis factor (TNF)-α was assessed using an RT-qPCR system (S100™ Thermal Cycler PCR machine, BIO-RAD, USA). Table S1 lists the specific primers used in this analysis. The 2^−ΔΔCT^ method was used to evaluate the data (Xu et al., 2020), with all values normalized to the expression of the housekeeping gene β-actin. The experiment was conducted three times to ensure reliability and reproducibility.

### 2.11 Temperature stability

Next, based on a previous study (Dong et al., 2021), we evaluated whether PAM-1 activity is affected by various factors. *E. coli* DC 8873 and DC 8466 were selected as the test strains to explore the effect of temperature. The prepared drug was incubated at different temperatures (−80°C, −20°C, 4°C, 25°C, and 37°C) for 3, 12, and 24 h. The MIC of PAM-1 was determined using the broth microdilution method after the incubation period.

### 2.12 Serum stability

Next, we evaluated the effect of serum on PAM-1 activity (Zhang et al., 2022). PAM-1 was incubated at 37°C for 24 h with various serum concentrations (0, 5, and 10%). After co-incubating the bacterial culture with serum, the MIC value of PAM-1 was evaluated using the same method mentioned above.

### 2.13 Ions stability

The effects of the presence of salts on the antimicrobial activity were evaluated as previously described (Kim et al., 2018). PAM-1 was incubated at 37°C with different concentrations of NaCl (0, 50, 100, and 150 mM), CaCl_2_ (0, 1.25, 2.5, and 5 mM) or MgCl_2_ (0, 0.5, 1, and 2 mM) for 24 h. Subsequently, the MIC of PAM-1 was determined using the broth microdilution method.

### 2.14 Cytotoxicity of PAM-1

Using RAW 264.7 cells, the safety of PAM-1 was assessed using a previously described method with some modifications (da Luz et al., 2020). The cell suspension (100 μL) containing 1 × 10^5^ cells was inoculated into the wells of a 96-well plate. Then, 10 μL of PAM-1 at various concentrations (1–256 μg/mL) was added to the medium. The plate was incubated at 37°C with 5% CO_2_ for 12 h. Thereafter, 10 μL of Cell Counting Kit-8 solution (CCK-8, Beyotime, China) was added to each well, followed by incubation at room temperature in the dark for 1 h. The absorbance of the wells was measured at 450 nm using the SpectraMax iD5 enzyme-labeled meter (BECKMAN, USA). This experiment was repeated three times.

### 2.15 Hemolysis of red blood cells (RBCs)

The hemolytic activity of PAM-1 was determined as previously described (Dong et al., 2012). Female BALB/c mice were used according to the Chinese National Standards for Laboratory Animals (GB 14925–2010). The Zhejiang Association for Science and Technology SYXK approved these analyses (ID: SYXK [Zhejiang] 2018–0017) and were consistent with the Wenzhou Laboratory Animal Welfare and Ethics standards. Fresh whole blood samples were collected and centrifuged. The RBCs obtained were washed with normal saline and then resuspended with normal saline to form a 5% RBC suspension. PAM-1 at various concentrations (1–80 μg/mL) was added to the 5% RBC suspension in a total volume of 2 mL. After centrifugation, the supernatant was collected and transferred to a 96-well plate. The absorbance of the supernatant was measured at 540 nm to reflect the hemolysis degree. The hemolysis rate was calculated using the following formula: Hemolysis rate (%) = (OD_experimental group_ − OD_negative control group_)/(OD_positive control group_ − OD_negative control group_), where OD represents the absorbance at 540 nm. The negative control group comprised only normal saline, whereas the positive control group comprised 0.1% Triton X-100. This experiment was performed three times.

### 2.16 *In vivo* effectiveness of PAM-1 in the *Galleria mellonella* model

The effectiveness of PAM-1 was evaluated *in vivo* by measuring the survival rate of the *G. mellonella* model, which is commonly used as a model organism for studying microbial infections (Tsai et al., 2016; Borjan et al., 2020). The larvae of *G. mellonella* used in this experiment were opalescent and weighed 250–300 mg. Overnight cultures of the bacteria were diluted to a density of 1.5 × 10^7^ CFU/mL. Then, the samples were divided into two groups: control and treatment groups. Furthermore, the treatment group was divided into subgroups, with each subgroup receiving different concentrations of PAM-1 (1/2MIC × 7, 1 MIC × 7, and 2MIC × 7). Using a microinjector, 10 μL of the bacterial suspension (CZA-susceptible *E. coli* DC 8873 and CZA-resistant *E. coli* DC 8466) was injected into the rear left proleg of each *G. mellonella* larva. After 2 h, the control group was administered 10 μL of sterile saline, whereas the treatment group received an appropriate concentration of the PAM-1 injection. To compare the efficacy of PAM-1 with standard agents, CZA was used. The larvae were then incubated at 37°C to measure their survival after 7 days. Larvae were considered dead if they repeatedly failed to respond to physical stimuli. The main experimental outcome was the analysis of the survival rate of *G. mellonella* using Kaplan–Meier analysis and the log-rank test.

### 2.17 Statistical analysis

All experiments were conducted in triplicate. Data analysis was performed using Prism 8 (GraphPad Software Inc., CA, USA), and the data are presented as mean and standard deviation. Significance was determined by using the two-sample *t*-test and log-rank test and indicated as *p < 0.05, ∗*p < 0.01, and ***p < 0.001.

## 3 Results

### 3.1 Physicochemical properties and determination of the antimicrobial activity of PAM-1

The properties and antibacterial activity of PAM-1 were determined using the Bioanalysis website. Table 1 presents the physical and chemical properties obtained via ProtParam analysis. PAM-1 comprises 34 amino acid residues, carries 9 net charges, and has an isoelectric point of 11.24. The GRAVY index of PAM-1 was −0.774 (negative value represents hydrophilicity), indicating that it exhibits good solubility.

**Table 1:** Analysis of the physicochemical properties of PAM-1.

Peptide	Net charge	Residue	PI	GRAVY	Molecular mass	Aliphatic index	Instability index
PAM-1	9	34	11.24	−0.774	3942.80	120.29	47.46

Next, the antibacterial activity of PAM-1 was predicted via biogenic analysis using CAMPR3. Both algorithms (Random Forest Classifier and Discriminant Analysis classifier) revealed that PAM-1 had better antibacterial activity (Table 2).

**Table 2:** Prediction of the antimicrobial activity of PAM-1.

Peptide	Residue	Random forest classifier	Discriminant analysis classifier
PAM-1	34	0.8235	0.991

### 3.2 MIC and MBC of PAM-1 against CZA-resistant *Escherichia coli*

Twelve nonrepetitive *E. coli* clinical isolates were selected. PCR was performed to determine the carbapenemase types of the experimental strains. Table 3 summarizes the MICs of PAM-1, CZA, and MEM and the MBC of PAM-1. These results suggest the effective antibacterial activity of PAM-1 against both CZA-resistant and susceptible *E. coli* strains. The MICs ranged from 2 to 8 μg/mL, whereas the MBCs ranged from 2 to 16 μg/mL. This indicates that PAM-1 exerts a bactericidal effect at the MBC.

**Table 3:** MICs of ceftazidime/avibactam, carbapenems, and PAM-1 and MBCs of PAM-1 against *Escherichia coli* and the carbapenemase types of strains.

Strains	MIC (μg/mL)					MBC (μg/mL)	Enzyme types
	PAM-1	CZA	MEM	IPM	ETP	PAM-1	
DC7914	4	>64/4^R^	32^R^	16^R^	>64^R^	4	NDM, TEM, CTX-M-9
DC8439	8	>64/4^R^	8^R^	4^R^	8^R^	8	NDM
DC8466	8	>64/4^R^	4^R^	4^R^	4^R^	8	NDM
DC8647	8	>64/4^R^	8^R^	8^R^	8^R^	16	NDM
DC8823	2	>64/4^R^	16^R^	16^R^	16^R^	2	NDM
DC10494	8	>64/4^R^	4^R^	4^R^	8^R^	8	NDM
DC8873	2	1/4^S^	0.25^S^	4^R^	<0.12^S^	8	TEM, SHV
DC10709	4	0.25/4^S^	1^S^	<0.12^S^	8^R^	8	CTXM-1, TEM, SHV
DC10740	4	0.5/4^S^	<0.12^S^	<0.12^S^	4^R^	16	CTX-M-9, SHV
DC11104	4	0.25/4^S^	2^I^	<0.12^S^	1^I^	16	CTX-M-1, TEM, SHV
DC11305	2	0.5/4^S^	<0.12^S^	<0.12^S^	<0. 12^S^	2	CTX-M-1, TEM, SHV
DC11308	8	0.5/4^S^	0.5^S^	0.5^S^	1^I^	16	CTX-M-1, TEM, SHV
ATCC25922	4	0.125/4^S^	0.06	0.25	0.015	4	/

### 3.3 PAM-1 inhibits bacterial growth

**Figure 1:**
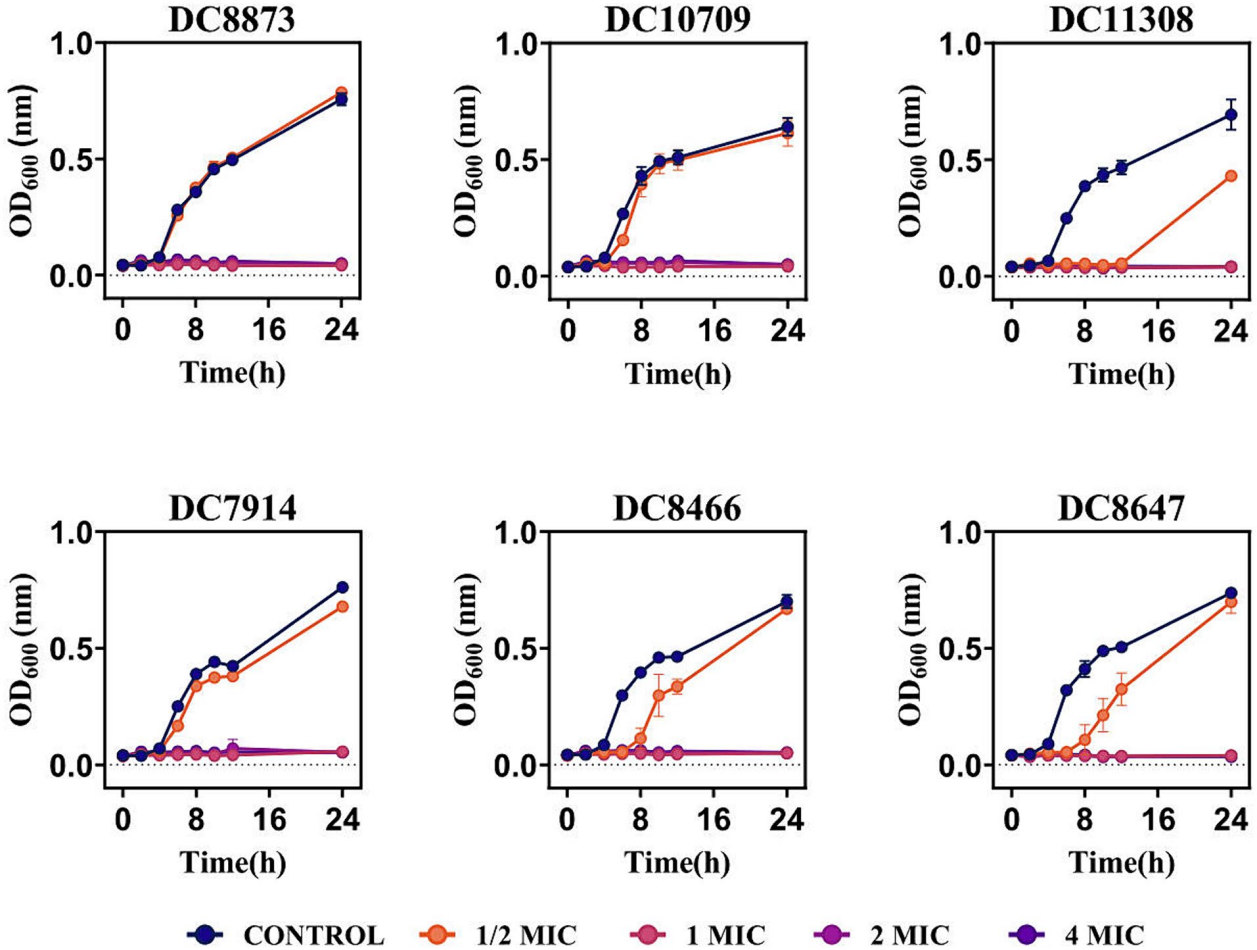
Growth curves of PAM-1 treatment against 3 CZA-susceptible *E. coli* and 3 CZA-resistant *E. coli*.

The effect of PAM-1 on bacterial growth was assessed by measuring OD_600_ changes over 24 h. Figure 1 demonstrates that the growth of the experimental strains was inhibited within 24 h when PAM-1 concentration exceeded or was equal to the 1 MIC. This finding suggests that PAM-1 exhibits a prolonged antibacterial effect on *E. coli* at the 1 MIC, and it even demonstrates a 12 h antibacterial effect on DC 11308 at 1/2 MIC concentration.

### 3.4 PAM-1 inhibits biofilm growth and eradicates The formed biofilms

**Figure 2:**
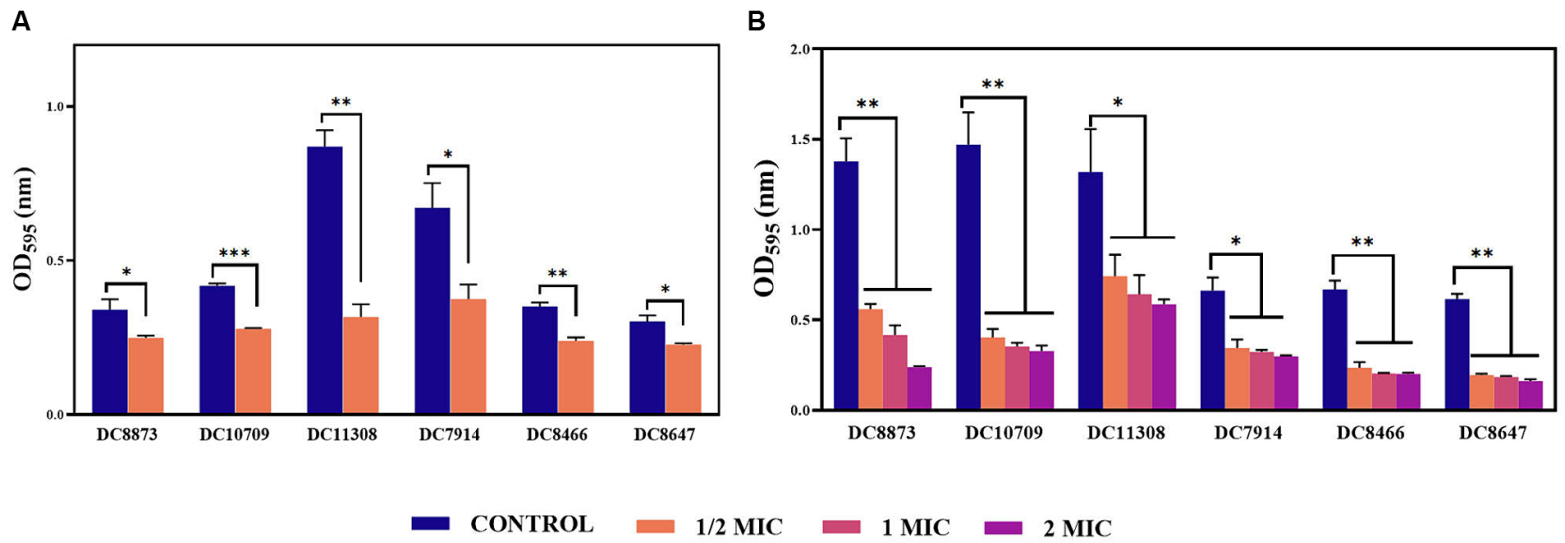
The effects of PAM-1 on *E. coli* biofilm. **(A)** The effect of PAM-1 on biofilm formation. **(B)** The effect of PAM-1 on the eradication of established biofilms. *p < 0.05, **p < 0.01, ***p < 0.001 were analyzed by Student’s *t*-test. The experiments were conducted thrice.

Crystal violet staining was used to determine the effect of PAM-1 treatment on inhibiting and eradicating biofilm formation. Figure 2A illustrates a noteworthy reduction in biofilm formation in the PAM-1-treated group compared to the control group. This observation suggests that1/2 MIC concentration PAM-1 possesses the ability to inhibit the formation of *E. coli* biofilm. Furthermore, it has an analogous efficacy in eradicating the pre-formed biofilms. Additionally, the eradication of established biofilms is achieved in a concentration-dependent manner, as illustrated in Figure 2B.

### 3.5 PAM-1 destroys bacterial and biofilm structures under SEM

**Figure 3:**
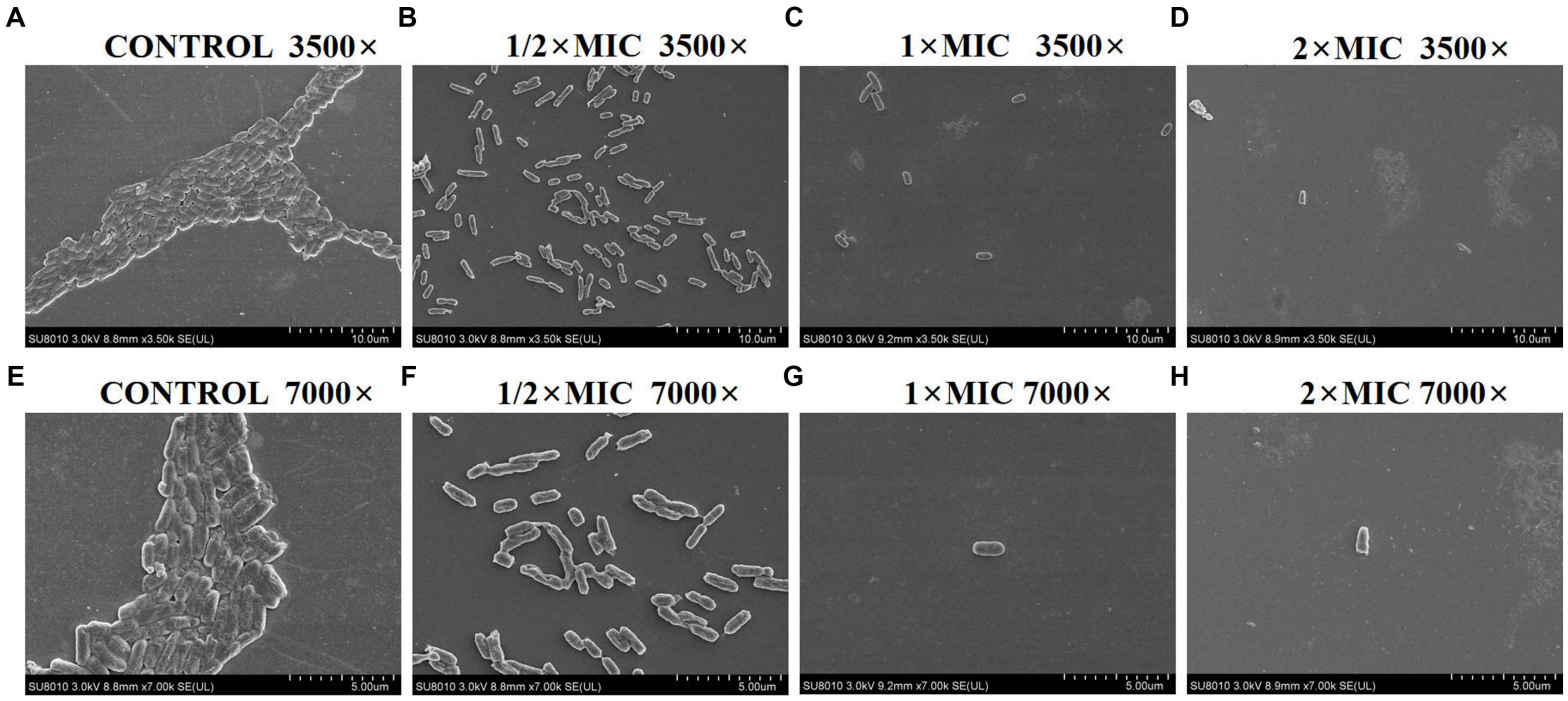
SEM images of *E. coli* DC 8466 biofilm and bacterial morphology results among various groups. **(A)** The broth control group, 3,500×, **(E)** 7,000×; **(B)** PAM-1 (1/2 MIC), 3,500×, **(F)** 7,000×; **(C)** PAM-1 (1 MIC), 3,500×, **(G)** 7,000×; **(D)** PAM-1 (2 MIC), 3,500×, **(H)** 7,000 ×.

SEM analysis revealed significant morphological changes of *E. coli* biofilm treated with various concentrations of PAM-1 as compared with the untreated control group (Figure 3). In detail, SEM images exhibited a fully formed and dense biofilm in the control group, with no noticeable bacterial destruction. However, a significant decrease in its number and density and changes in its structure were observed after PAM-1 treatment. And some bacteria also showed damage to some extent.

### 3.6 PAM-1 increases the permeability of bacterial membranes

**Figure 4:**
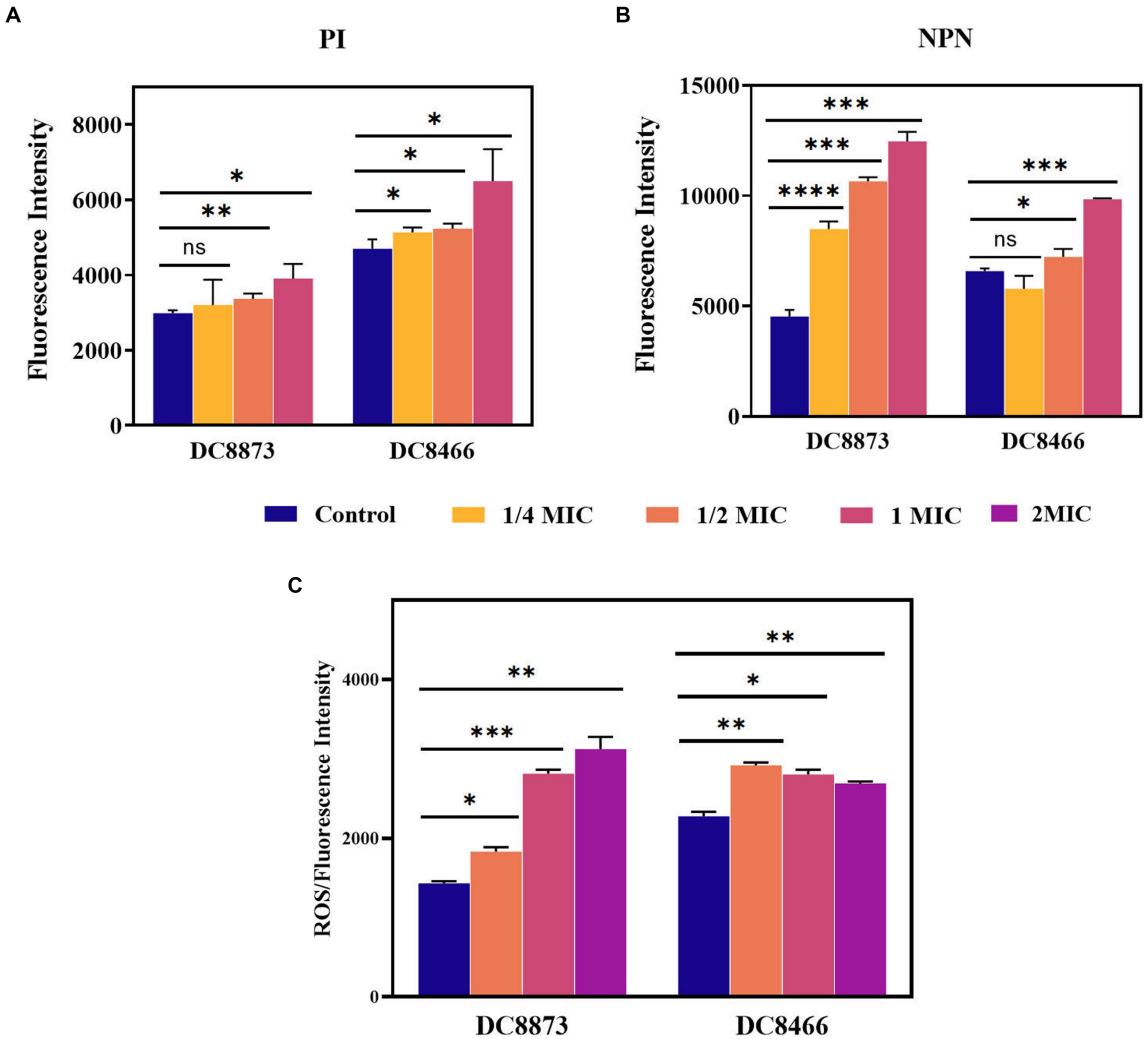
The antimicrobial mechanism of PAM-1 was investigated by PI **(A)**, NPN **(B)**, and **(C)** ROS. The fluorescence intensity of DC 8873 and DC 8466 treated with different concentrations of PAM-1. ns, not statistically significant; *p < 0.05, **p < 0.01, ***p < 0.001, ****p < 0.0001.

Figure 4 illustrates that PAM-1 increased bacterial membrane permeability. To investigate the changes in cell membrane permeability induced by PAM-1, two different assays were performed: PI staining and NPN uptake assay. PI is a probe that cannot penetrate the cell membrane and can only bind to the nucleic acid of bacteria with damaged membranes. On the other hand, NPN can help assess the permeability of the outer membrane. Normally, NPN is hindered by the intact outer membrane. However, if the outer membrane structure is compromised and its permeability is altered, NPN can enter the interior of the cell and emit fluorescence. Both PI staining and the NPN uptake assay revealed that the fluorescence intensity of PAM-1 treated group was significantly higher than that of untreated group, indicating that PAM-1 induced a dose-dependent increase in membrane permeability.

Furthermore, PAM-1 significantly increased ROS levels (Figure 4C); this may have played a role in its antibacterial activity. The level of ROS is closely correlated with the physiological activities of cells. When intracellular ROS production surpasses the cells’ natural antioxidant defenses, it induces oxidative stress, resulting in cellular damage and apoptosis (Azad et al., 2009). Collectively, these findings suggest that even at low concentrations, PAM-1 can effectively damage bacterial membranes, increase cell membrane permeability, and ultimately lead to bacterial death.

### 3.7 PAM-1 decreases the expression of inflammatory cytokines

**Figure 5:**
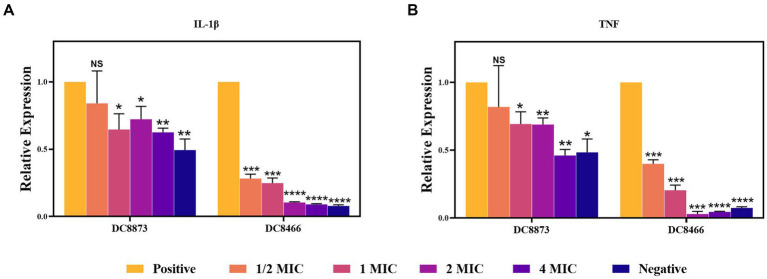
The effect of PAM-1 on RAW 264.7 inflammatory factor after *E. coli* infection. Negative, only cells are added; Positive, strain and cell co-infection (without PAM-1); NS, not statistically significant; *p < 0.05, **p < 0.01, ***p < 0.001, ****p < 0.0001.

RT-qPCR was performed to elucidate the anti-inflammatory effects of PAM-1. Initially, using a cytotoxicity assay, we confirmed that an MOI of 10 did not affect cell viability. Therefore, RAW 264.7 cells were infected with bacterial cultures with an MOI of 10; then, the expression of IL-1β and TNF-α was analyzed. Figure 5 demonstrates that DC 8873 and DC 8466 significantly promoted IL-1β and TNF-α expression compared with the negative control group. However, PAM-1 dose-dependently inhibited IL-1β and TNF-α expression. The effect was more pronounced in DC 8466, which exhibited more significant inhibition of inflammatory cytokine. This indicates that PAM-1 possesses anti-inflammatory properties.

### 3.8 Stability of PAM-1

We investigated the stability of PAM-1 under different conditions, including changes in temperature, serum exposure, and presence of salt ions. Table 4 demonstrates that neither ambient nor low temperatures affected the antibacterial activity of PAM-1. Furthermore, based on the provided instructions, it can be stored at −20°C for extended periods. Moreover, we observed that PAM-1 maintained significant antibacterial activity even in the presence of 10% serum (Table 5). Unfortunately, PAM-1 exhibited poor performance in a saline environment, with the presence of NaCl, CaCl_2_, and MgCl_2_ considerably decreasing its antibacterial efficacy (Table 6). This phenomenon could be attributed to the initial interaction of the positively charged cationic peptide as it interacts with and neutralizes the negatively charged bacterial outer membrane through electrostatic interaction (Bin Hafeez et al., 2021). Moreover, the presence of salt ions not only establishes a salt bridge with lipopolysaccharides to stabilize the outer membrane but also leads to the need for higher concentrations of AMPs to compete with and replace the salt ions on the outer membrane.

**Table 4:** The effect of temperature on the antibacterial activity of PAM-1 [MIC (μg/mL)].

**Temperature (°C)**	Bacacterial isolates					
	DC8873			DC8466		
	3 h	12 h	24 h	3 h	12 h	24 h
**−80**	2	2	2	8	8	8
**−20**	2	2	2	8	8	8
**4**	2	2	2	8	8	8
**25**	2	2	2	8	8	8
**37**	2	2	2	8	8	8

**Table 5:** The effect of serum on the antibacterial activity of PAM-1 [MIC (μg/mL)].

Serum concentration (%)	Bacterial isolates	
	DC8873	DC8466
0	2	8
5	2	8
10	2	8

**Table 6:** The effect of conditions mimicking physiological concentrations of salt on the antibacterial activity of PAM-1 [MIC (μg/mL)].

Salt	Concentration	Bacterial isolates	
		DC8873	DC8466
NaCl	0 mM	2	8
	50 mM	4	32
	100 mM	16	32
	150 mM	16	>128
CaCl_2_	0 mM	2	8
	1.25 mM	4	16
	2.5 mM	4	16
	5 mM	4	16
MgCl_2_	0 mM	2	8
	0.5 mM	>128	>128
	1 mM	>128	>128
	2 mM	>128	>128

### 3.9 Safety of PAM-1

**Figure 6:**
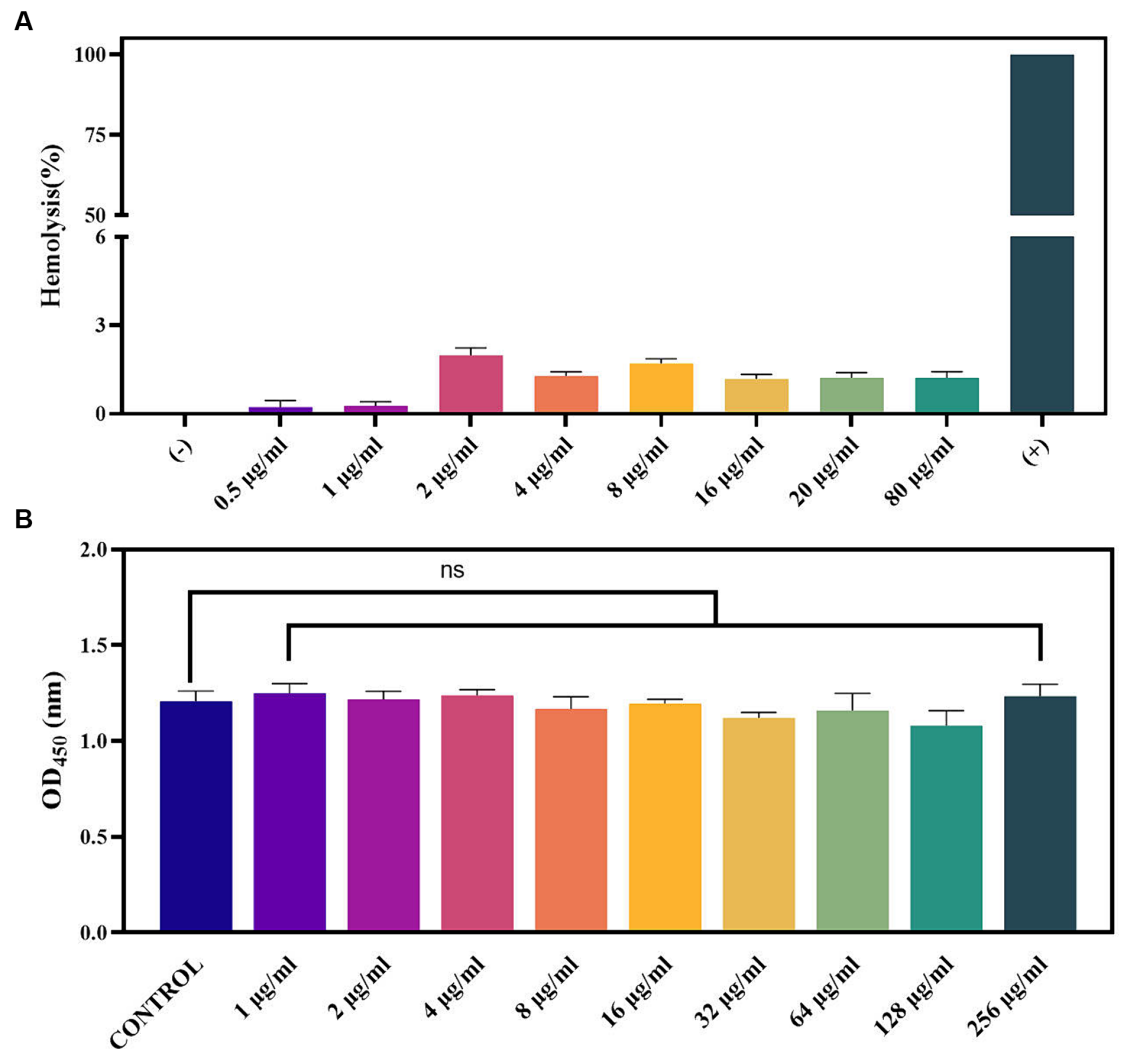
Toxicity of PAM-1. **(A)** The effect of PAM-1 on erythrocytes; **(B)** cytotoxicity of PAM-1 with different concentrations against RAW 264.7 murine macrophage cell line. ns, not statistically significant.

To further investigate the security of PAM-1, RBC hemolysis and cytotoxicity were assessed (Figure 6). The hemolysis rate was <3% even when PAM-1 concentration reached 80 μg/mL; furthermore, no visible hemolysis was observed (Figure 6A). In addition, PAM-1 exhibited no cytotoxicity in RAW 264.7 cells at a concentration of 256 μg/mL (Figure 6B). Collectively, these findings suggest that PAM-1 holds promising prospects for *in vivo* applications.

### 3.10 PAM-1 can improve the survival rate of *Galleria mellonella*

**Figure 7:**
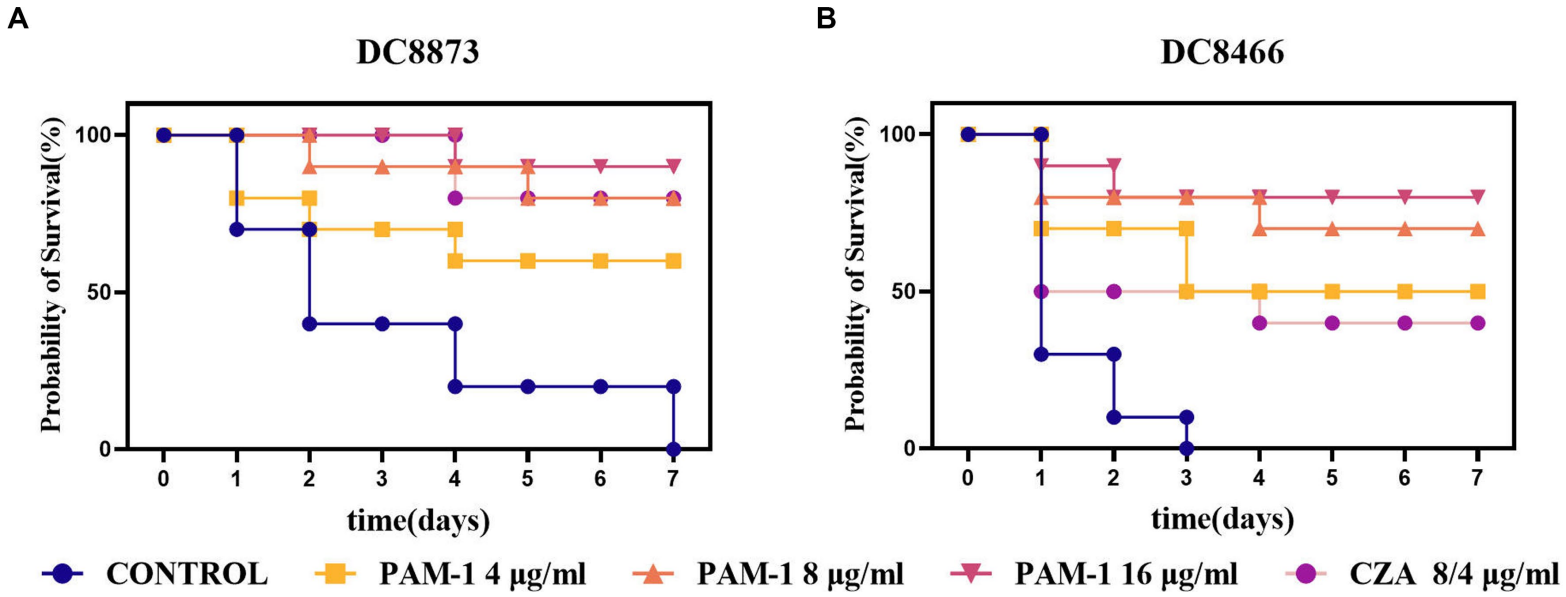
The survival rate of *G. mellonella* for different therapies. DC 8873 and DC 8466 as the experimental strains, and the survival rate record of *G. mellonella* in 7 days.

Considering the findings from *in vitro* antibacterial studies, conducting *in vivo* experiments is vital to confirm the antibacterial activity of PAM-1 in living organisms. To this end, a *G. mellonella* infection model was developed to assess the *in vivo* bactericidal activity of PAM-1 and compare it with that of CZA (Figure 7). Compared with the control group, PAM-1 significantly improved the survival rate of *G. mellonella*. After 7 days of PAM-1 therapy, the survival rates of DC 8873 (CZA-susceptible *E. coli*) and DC 8466(CZA-resistant *E. coli*) were > 60 and > 50%, respectively; however, all control subjects died at 7 and 4 days, respectively. Importantly, the survival rate of the PAM-1 treatment groups was either comparable with or higher than that of the CZA treatment groups; this suggests the superior efficacy of PAM-1 over CZA. Collectively, these results suggest the potential efficacy of PAM-1 in treating *E. coli* infections.

## 4 Discussion

Owing to the worldwide emergence of antibiotic resistance and the declining rate of new antibiotic discovery, exploring novel antimicrobial agents has become an urgent issue. The increase in multidrug-resistant microorganisms poses a challenge for doctors and leads to higher rates of infections and deaths among seriously ill patients (Peleg and Hooper, 2010; Cornejo-Juárez et al., 2015). AMPs have been identified as promising candidates for antimicrobial therapy. They exert broad-spectrum antimicrobial activity and anti-inflammatory effects, have a rapid mode of action, and have minimal drug resistance development in pathogens (Yeaman and Yount, 2003).

AMPs derived from various natural sources, including plants, animals, and humans, provide valuable compositional and structural information. For example, WAM-1 from marsupials exhibits broad-spectrum antimicrobial activity (Wang et al., 2011); furthermore, AMPs from scorpion venom can kill multidrug-resistant pathogens (Harrison et al., 2014; Hong et al., 2021). However, some AMPs have inherent limitations, including low abundance, cytotoxicity, and instability under some conditions (Mahlapuu et al., 2020).

Although the *in vitro* antibacterial activity of PAM-1, an AMP isolated from the platypus, against standard strains of *B. subtilis*, *S. aureus*, and *E. coli* has been studied (Wang et al., 2011), studies on clinical strains and further assessment of its antibacterial effects and mechanisms in both *in vitro* and *in vivo* settings are lacking. Therefore, in the present study, we elucidated the antibacterial, antibiofilm, and anti-inflammatory activities of PAM-1 and its underlying mechanisms as well as evaluated its safety and efficacy *in vivo*.

First, PAM-1 exhibited significant antibacterial activity against both CZA-sensitive and resistant *E. coli* isolates (Table 3). PI staining and the NPN uptake assay revealed that PAM-1 may kill bacteria by destroying the bacterial membrane (Figure 4). Previous studies have reported that positively charged PAM-1 may disrupt the integrity of the bacterial membrane and exert antibacterial effects by interacting with and neutralizing the negatively charged bacterial outer membrane via electrostatic interactions, thereby forming transmembrane pores via the “barrel-stave” model (Oren and Shai, 1998; Bin Hafeez et al., 2021; Han et al., 2022). Based on this mechanism of action, PAM-1 can sustain antibacterial effects over an extended period, making it a promising therapeutic agent. During aerobic metabolism, cells continuously produce ROS, and protect against ROS overproduction. However, oxidative stress is induced when ROS production overwhelms the natural antioxidant defenses of the cell. PAM-1 may induce bacterial cell damage and apoptosis by significantly increasing ROS levels (Figure 4).

Biofilms are microbial communities attached to solid surfaces; they enhance bacterial adaptability to the environment, impede the penetration of conventional antibiotics, and contribute to recurrent infections (Longo et al., 2014; Flemming et al., 2016). PAM-1 exhibited a notable inhibitory effect on biofilm growth and mature biofilm eradication (Figure 2). SEM analysis revealed that PAM-1 effectively destroys biofilms and inflicts significant damage to the bacteria (Figure 3).

Inflammatory responses play a vital role in defending against pathogens (Kaczyńska et al., 2016). For example, TNF-α actively participates in the pathogenesis of skin infections (Mittal et al., 2010). Bacterial infections often trigger inflammation and associated complications, making it necessary to regulate proinflammatory factor production. RT-qPCR revealed that PAM-1 can effectively alleviate inflammatory responses by decreasing IL-1β and TNF-α production (Figure 5).

To evaluate the clinical potential of PAM-1, cytotoxicity and RBC hemolysis assays were conducted (Figure 6). Unlike most AMPs, PAM-1 maintained its antibacterial activity at 37°C as well as in the presence of serum (Tables 4, 5). However, Table 6 indicate PAM-1 exhibited instability in the presence of salt ions. This may be because cationic AMPs initially interact with and neutralize the negatively charged bacterial outer membrane via electrostatic interactions (Bin Hafeez et al., 2021). The presence of salt ions not only stabilizes the outer membrane by establishing a salt bridge with lipopolysaccharides but also increases the demand for AMP to replace the salt ions on the outer membrane (Han et al., 2022). Therefore, additional studies are warranted to explain the competition and stabilization mechanisms between AMP and salt ions and to solve the application of cationic AMPs in high-salt environments.

Based on the findings of the abovementioned experiments, we used the *G. mellonella* larval infection model to further verify the *in vivo* therapeutic effect of PAM-1. Compared with the treatment effect of CZA, the survival rate of *G. mellonella* larvae treated with PAM-1 was similar or better (Figure 7). This suggests that PAM-1 had a good application prospect *in vivo* and can exert a better therapeutic effect than CZA *in vivo*.

**Figure 8:**
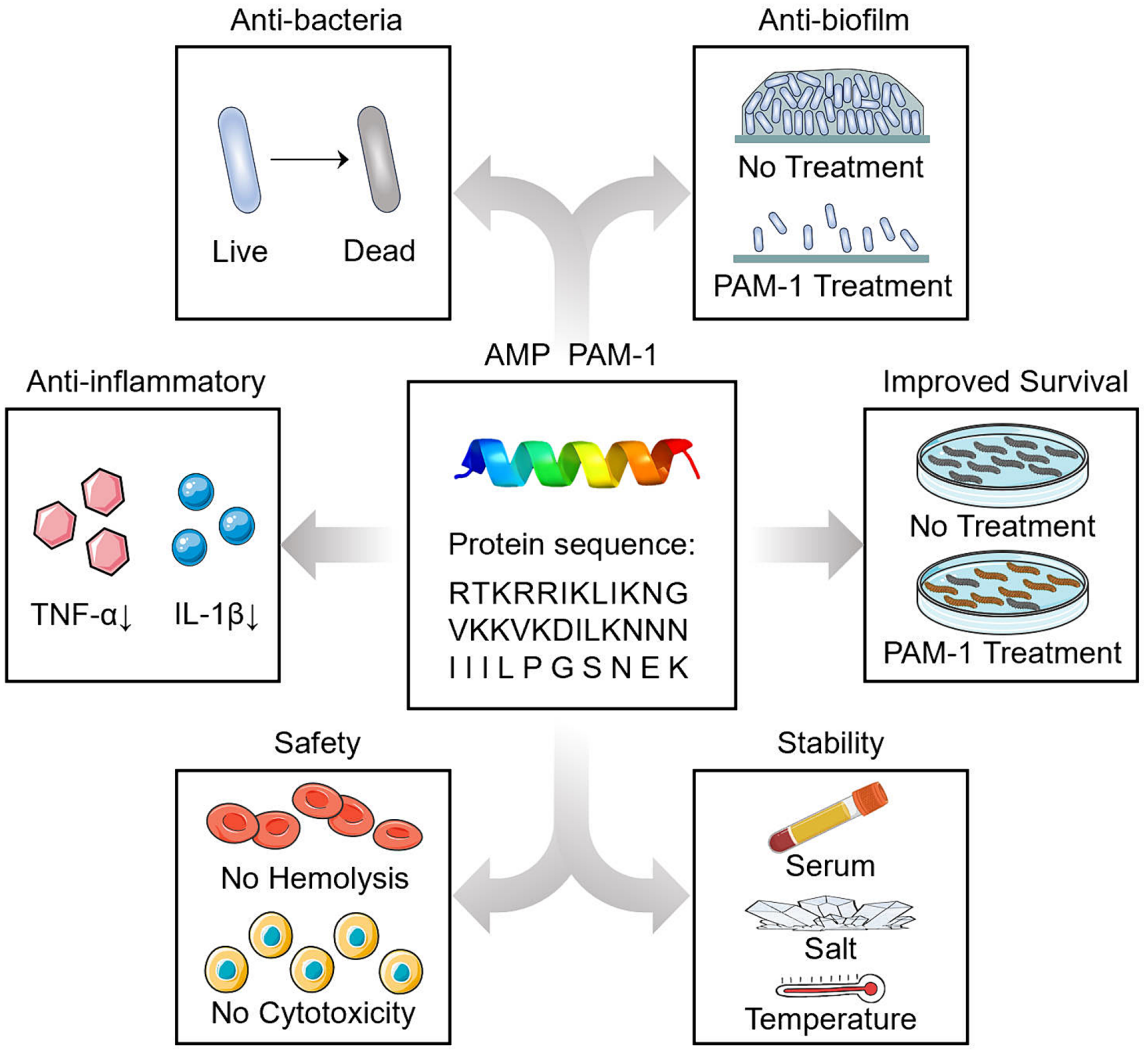
Summary diagram.

This is the first study to demonstrate the antibacterial effect of PAM-1 against CZA-resistant *E. coli* both *in vitro* and *in vivo*. We observed that PAM-1 is a promising agent for treating CZA-resistant *E. coli* infections. In addition to antibacterial and antibiofilm activities, PAM-1 also exhibits potent anti-inflammatory effects (Figure 8). Although the mechanism underlying the effect of salt ions on its activity deserves further investigation, PAM-1 exhibits good stability and effects in serum, at room temperature, and *in vivo*. Collectively, these findings indicate that PAM-1 is a promising antibacterial agent against drug-resistant *E. coli* infections.

## Data availability statement

The original contributions presented in the study are included in the article/Supplementary material, further inquiries can be directed to the corresponding authors.

## Ethics statement

The animal study was approved by the Zhejiang Association for Science and Technology SYXK. The study was conducted in accordance with the local legislation and institutional requirements.

## Author contributions

YH: Data curation, Formal analysis, Investigation, Methodology, Software, Writing – original draft. YZ: Investigation, Methodology, Validation, Writing – review & editing. XZ: Conceptualization, Supervision, Writing – review & editing. ZH: Investigation, Software, Writing – review & editing. JK: Investigation, Writing – review & editing. XW: Investigation, Project administration, Resources, Writing – review & editing. LC: Investigation, Resources, Writing – review & editing. YW: Methodology, Software, Supervision, Validation, Writing – review & editing. JC: Conceptualization, Data curation, Supervision, Writing – review & editing. TZ: Conceptualization, Funding acquisition, Resources, Supervision, Writing – review & editing. MS: Conceptualization, Resources, Supervision, Writing – review & editing.
