# Supplementary material for: PAM-1: an antimicrobial peptide with promise against ceftazidime-avibactam resistant Escherichia coli infection
Source: Front Microbiol. 2024 Apr 30;15:1291876. doi: 10.3389/fmicb.2024.1291876 (PMC11099939; doi:10.3389/fmicb.2024.1291876)
Supplement: Supplementary file 1 [file Table_1.DOCX]

PAM-1: An antimicrobial peptide with promise against ceftazidime-avibactam resistant *Escherichia coli* infection

**Yijia Han^1，2^, Yi Zhang^2^, Xiaodong Zhang^1^, Zeyu Huang^1^ Jingchun Kong^2^, Xiuxiu Wang^1^, Lijiang Chen^1^, Yue Wang^1^, Jianming Cao^2^，Tieli Zhou^1*^, Mo Shen^1*^**

^1^ Department of Clinical Laboratory, The First Affiliated Hospital of Wenzhou Medical University; Key Laboratory of Clinical Laboratory Diagnosis and Translational Research of Zhejiang Province

^2^ School of Laboratory Medicine and Life Science, Wenzhou Medical University, Wenzhou, China.

*** Correspondence:**Tieli Zhou,

wyztli@163.com

Mo Shen,

shenmo601@163.com

Keywords: *E. coli*, biofilm, CZA -resistant, PAM-1, antimicrobial peptide

| **Table S1**. Primer sequence, production size, and annealing temperature used in this study | | | | |
| --- | --- | --- | --- | --- |
| **Primer** | **Sequence** | **Size(bp)** | | **Annealing(℃)** |
| *bla*_KPC_ | **F:** AAGATCTACAACCACAGCATTC **R:** CAGACTCCTAGCCTAAATGTGA | | 1381 | 55 |
| *bla*_NDM_ | F: GGTTTGGCGATCTGGTTTTC  **R: CGGAATGGCTCACGATC** | | 621 | 52 |
| *bla*_IMP_ | F: CATGGTTTGGTGGTTCTTGT  **R: ATAATTTGGCGGACTTTGGC** | | 488 | 50 |
| *bla*_VIM_ | **F: GATGGTGTTTGGTCGCATA**  **R: CGAATGCGCAGCACCAG** | | 390 | 58 |
| *bla*_OXA-23_ | **F: ACTTGCTATGTGGTTGCTTCTCTT**  **R: TTCAGCTGTTTTAATGATTTCATCA** | | 797 | 55 |
| *bla*_OXA-48_ | **F:TTGGTGGCATCGATTATCGG**  **R:GAGCACTTCTTTTGTGATGGC** | | 744 | 55 |
| *bla*_SHV_ | **F: AGCCGCTTGAGCAAATTAAAC**  **R: ATCCCGCAGATAAATCACCAC** | | 713 | 60 |
| *bla*_TEM_ | **F: CATTTCCGTGTCGCCCTTATTC**  **R: CGTTCATCCATAGTTGCCTGAC** | | 800 | 60 |
| *bla*_CTX-M-1_ | **F:AAAAATCACTGCGTCAGTTCAC**  **R: ACAAACCGTTGGTGACGATT** | | 867 | 55 |
| *bla*_CTX-M-9_ | **F: TATTGGGAGTTTGAGATGGT**  **R: TCCTTCAACTCAGCAAAAGT** | | 933 | 50 |
| *bla*_CTX-M-14_ | **F: CTGCTTAATCAGCCTGTCGA**  **R: TCAGTGCGATCCAGACGAAA** | | 230 | 50 |
| IL-1β | **F: GAAATGCCACCTTTTGACAGTG R:** TGGATGCTCTCATCAGGACAG | | 116 | 50 |
| TNF-α | F: ATGAGAAGTTCCCAAATGGC  **R: CTCCACTTGGTGGTTTGCTA** | | 125 | 50 |
